# Psychological Therapy in Chronic Pain: Differential Efficacy between Mindfulness-Based Cognitive Therapy and Cognitive Behavioral Therapy

**DOI:** 10.3390/jcm10163544

**Published:** 2021-08-12

**Authors:** Estela María Pardos-Gascón, Lucas Narambuena, César Leal-Costa, Antonio Jesús Ramos-Morcillo, María Ruzafa-Martínez, Carlos J. van-der Hofstadt Román

**Affiliations:** 1 Sexual and Reproductive Health Unit, Novelda Health Center, 03660 Alicante, Spain; estela.maria.pardos.gascon@gmail.com; 2 Child-Youth Mental Health Unit, Can Misses Hospital, 07800 Ibiza, Spain; narambuenalucas@gmail.com; 3 Faculty of Nursing, University of Murcia, 30100 Murcia, Spain; maruzafa@um.es; 4 Hospital Psychology Unit, Department of Health Psychology, Institute of Health and Biomedical Research of Alicante (ISABIAL), General University Hospital of Alicante, Miguel Hernández University, 03010 Alicante, Spain; cjvander@umh.es

**Keywords:** chronic pain, MBCT, CBT, differential efficacy, effect sizes

## Abstract

The objective of this study is to evaluate the differential efficacy between Mindfulness-Based Cognitive Therapy (MBCT) and Cognitive Behavioral Therapy (CBT). A quasi-experimental design of repeated measures before and after the test (*n* = 57) was used with a non-equivalent control group from a previous cohort treated with CBT (*n* = 105). The *t*-test revealed significant differences in subjective quality of life for the MBCT group, and in quantity, optimum, and adequate sleep for the CBT group. The pre–post effect size comparison mostly showed slightly larger effect sizes in the MBCT group. CBT and MBCT had comparable efficacies, although a slight trend towards larger effect sizes in MBCT was found. Likewise, CBT seemed to improve sleep-related variables, while MBCT was associated with improvements in pain and quality of life.

## 1. Introduction

Non-oncological chronic pain is defined as pain that persists for more than 3 months after the occurrence of a lesion, in the absence of any oncological process [1]. Its prevalence has been calculated to be 20% worldwide [2], and it is a medical condition with a high economic burden due to its relationship with work absenteeism, a decrease in productivity, early retirement, and work disability, with its indirect and direct costs in the European Union estimated at 3 to 10% of the gross domestic product [3].

Given its multi-factorial character, pharmacological treatment tends to be insufficient, and therefore, multidisciplinary approaches are indispensable [4]. In this sense, the psychological treatment of choice for chronic pain is Cognitive Behavioral Therapy (CBT) [5]. However, despite the considerable amount of evidence in its favor, research studies have only focused on a limited number of pathologies, and effect sizes tend to be small [6]. In parallel, treatments for chronic pain based on Mindfulness have also been developed, such as Mindfulness-Based Stress Reduction (MBSR) [7] and Mindfulness-Based Cognitive Therapy for Chronic Pain (MBCT-CP) [8]. A recent transdiagnostic study [9] and several meta-analyses [10,11,12] have indicated that although MBCT-CT is superior to CBT, in general, it has a similar efficacy in variables related to pain intensity and interference, as well as psychological variables, in all diagnostic groups (fibromyalgia, low back pain, unspecified chronic pain and headache) [10,11,12]. However, studies on the subject are scarce and highly heterogeneous concerning the variables studied, the types of samples, and the measurement instruments used. Thus, the establishment of solid evidence and the identification of differences in efficacy between treatments have been difficult.

The objective of the present work is to evaluate the differential efficacy between MBCT-CP and CBT in a multi-center sample of patients diagnosed with different medical conditions associated with non-oncological chronic pain in the province of Alicante, Spain.

## 2. Materials and Methods

### 2.1. Design

Quasi-experimental design of repeated measures before and after the test (*n* = 57), with a non-equivalent control group from a previous cohort.

### 2.2. Participants

To achieve a power of 80% in the detection of significant differences in a two-tailed Student’s *t*-test for two related samples, with a significance level of 5%, assuming a 50% reduction in pain from the first basal measurement to the last visit, and with a standard deviation of 8, the need for 40 sets of records was calculated, assuming a 15% loss.

Thus, between June 2018 and January 2019, a sample of 57 participants was obtained through a convenient, non-randomized sample obtained from the waiting lists at the pain units of three hospitals located in the province of Alicante: General University Hospital of Alicante (GUHA), Marina Baixa Hospital (MBH) (Villajoyosa), and the Vega Baja Hospital (VBH) (Orihuela). The same sampling method was utilized at the pain unit at the GUHA, where Cognitive Behavioral Therapy for chronic pain was implemented between March 2011 and February 2015. This sample was constituted of the non-equivalent control group from a previous cohort (*n* = 105).

The inclusion criteria for both groups were: (1) being older than 20 years old, without an upper age limit, (2) non-oncological chronic pain lasting for more than 3 months and with a moderate-severe intensity diagnosed by doctors from the pain unit, (3) with this pain interfering in at least one area of daily activity, (4) having the autonomy necessary for filling out the self-administered tests, and (5) acceptance to participate in the study with a written informed consent form. The following subjects were excluded: (1) those with decreased capacity for providing informed consent, and (2) those with a severe clinical comorbidity (for example: personality disorder, major depression), or (3) with an acute-phase clinical comorbidity.

### 2.3. Instruments

For both groups, a notebook was created with the following questionnaires:

- Sociodemographic characteristics: age, sex, marital status, occupation.
- Self-reported pain intensity: using the Visual Analogue Scale (VAS) [13] (0 = absence of pain, 10 = worst pain imaginable), pain was assessed in the 3 days prior to the study. The VAS has reported convergent validity values ranging from 0.30 to 0.95, and moderate concurrent validity (0.71–0.78) when compared with the Numeric Pain Rating Scale, with high test–retest reliability (ICC = 0.71–0.99) [14,15,16,17]. For the assessment of pain at the present time, a Likert scale with 4 response options was utilized (0 = no pain, and 4 = extremely intense).
- Anxiety/depressive symptoms: the Hospital Anxiety and Depression Scale (HADS) [18], validated in a Spanish population with chronic pain [19] with adequate reliability (anxiety: α = 0.83; depression: α = 0.87), is comprised of 14 self-assessed items, using a Likert response scale with 4 options. Its global scores oscillate between 0 and 42, and for each subscale, from 0 to 21.
- Perception of state of health: the SF-12 state of health scale (Short Form SF-12) [20] is comprised of 12 items extracted from the SF-36, with Likert response options ranging from 3 to 6 points. A measurement of overall physical and mental health is obtained, with scores ranging from 0 (worst state of health) to 100 (best state of health). Its reliability was shown to be adequate (physical overview: α = 0.85; mental overview: α = 0.78) [21].
- The interference of sleep by pain: using the 12 items from the Medical Outcomes Study Sleep Scale (MOS Sleep Scale) [22], the impact of external stimuli on attributes of sleep architecture (adequacy, optimum sleep, quantity, abrupt awakenings, snoring, altered sleep, and somnolence) was explored, as well as the overall sleep interference index with 6 and 9 items, with responses ranging from 0 (no interference) to 100 (maximum interference). The scale showed good reliability (α = 0.64–0.87) for patients with neuropathic pain [23].
- Perception of self-efficacy in the management of pain: The Chronic Pain Self-Efficacy Scale was selected [24], which showed adequate reliability according to its authors (control of symptoms: α = 0.85, physical functioning: α = 0.98; management of pain: α = 0.72, total self-efficacy score: α = 0.91). It is composed of 19 items with Likert-types responses of 10 points, with a higher score indicating a greater degree of self-efficacy.

### 2.4. Procedure

In both groups, the patients on the waiting lists from each pain unit were divided into groups composed of 10 to 12 participants. The questionnaires were completed in the first and last sessions.

In the experimental group, Mindfulness-Based Cognitive Therapy for Chronic Pain (MBCT-CP) was administered [8], composed of 8 weekly group sessions that lasted an hour and a half each (see Table 1). Four groups were created at the GUHA (*n* = 23), 2 groups at the MBH (*n* = 11), and 2 groups at the VBH (*n* = 23) between September 2018 and June 2019. The research authors translated the patient materials provided during therapy from the MBCT-CP [8]. In the control group, a CBT protocol created for chronic pain at the General Hospital of Alicante [25] was utilized. It consisted of 8 weekly sessions lasting 120 min each (see Table 1).

Both interventions were performed by clinical psychology residents with experience in group therapy, but without specific knowledge of Mindfulness or CBT for chronic pain. MBCT-CP was provided by 6 residents and CBT for chronic pain by 4.

### 2.5. Confidentiality

The collection, treatment, and use of the data required by this study was performed in agreement with the Organic Law 15/1999 of protection of personal data and its development guidelines, in this case Royal Decree 1720/2007, as well as Regulation 2016/679 of the European Parliament and Council, dated 27 April 2016, related to the treatment of personal data, and any applicable norms and/or legislation. Likewise, the research project for the application of Mindfulness-Based Cognitive Therapy protocol was approved by the Ethics Committee for Research with medicines (CEIm) from the GUHA (Ref.CEIm: PI2018/109 Ref.ISABIAL: 180296), and the Cognitive Behavioral Therapy was approved by the Ethics Committee from the GUHA (CEIC PI2011/51).

### 2.6. Statistical Analysis

The descriptive statistics were analyzed with IBM SPSS Statistics^®^ v.24 (IBM, Armonk, NY, USA), depending on the nature of the variables, as well as with Student’s *t*-test for dependent and independent samples, after verification of normality (Kolmogorov–Smirnov test) and homoscedasticity (Levene’s test). Complementarily, effect size was analyzed through Cohen’s d, with a d between 0.2 and 0.49 considered “small”, a d between 0.5 and 0.79 as “medium”, and a d greater than 0.8 as “large” [26].

## 3. Results

### 3.1. Characteristics of Sample and Pretreatment Differences

**Figure 1:**
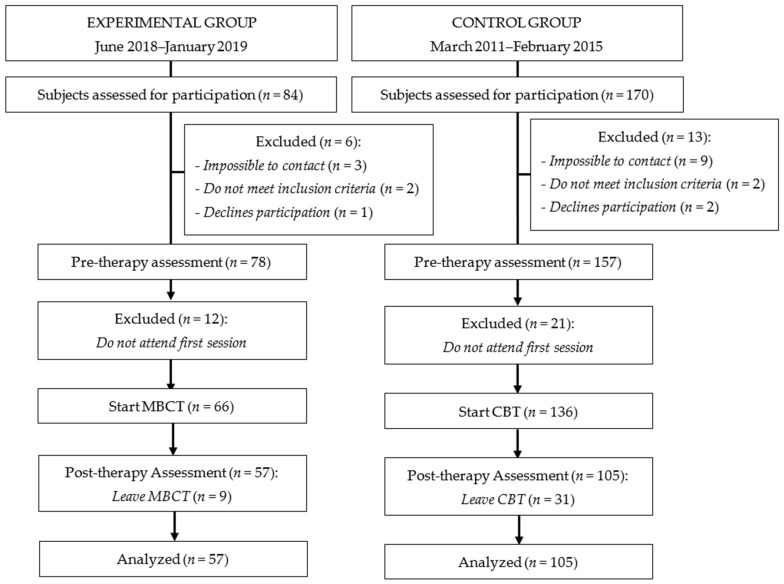
Flow diagram of participants.

In the experimental group, 84 participants started the program, with 57 of them finishing it (for a response rate of 67.85%). Likewise, in the control group, the response rate was 61.76% (see Figure 1).

In the experimental group, most patients (38.6%) had a diagnosis of low back pain/lumbosciatic pain, followed by neck pain or cervicobrachialgia and fibromyalgia (12.3%), while 23.4% had a combination of chronic pains as their common characteristic. This diagnostic analysis could not be performed with the control group, as only the presence of fibromyalgia was recorded (16.2%).

As for the center, in the experimental groups, 40.4% were GUHA patients, with the same percentage from the VBH, while MBH patients were the least numerous (19.3%). The control group was entirely composed of GUHA patients.

To explore the differences between the experimental and control groups, the frequencies of sociodemographic variables were compared (sex, employment, marital status), as well as the presence of fibromyalgia, and age. The Chi-square test revealed the frequencies in all of these variables were balanced, except for the employment or occupation of the control groups, which showed a higher percentage (32.4%) of people with a disability (see Table 2), and age, where a Student’s *t*-test for independent samples indicated significant differences between the groups (*t* = −2.09, *p* = 0.04), with the average age of the experimental group being higher (M = 55.72, SD = 10.19) as compared to the controls (M = 51.98, SD = 11.06).

Likewise, the means before treatment were compared in both groups by Student’s *t*-test for independent samples, after verifying assumptions of normality (Kolmogorov–Smirnov test) and homoscedasticity (Levene’s test). Both groups showed very homogeneous values in all variables. However, statistically significant differences were found in some variables such as a greater self-efficacy in pain control (*t* = 2.00, *p* = 0.04), less adequate sleep (*t* = 8.12, *p* = 0.00), and more sleepiness (*t* = 3.91, *p* = 0.00) and sleep disturbance (*t* = 4.51, *p* = 0.00) in the control group, while the experimental group showed greater pain at the present time (*t* = −3.15, *p* = 0.00) (see Table 3). The sleep variable “optimum”, due to its nominal character, was analyzed through the Chi-square test, with no significant differences found.

### 3.2. Post-Treatment Differences

In the comparison of post-treatment results between the control and experimental groups, no significant differences were found for any variable, except for the experimental group, which showed a higher score for subjective quality of life (*t* = −3.34, *p* = 0.00), and the control group, with a statistically significant improvement in quantity of sleep (*t* = 2.53, *p* = 0.01), and adequate sleep (*t* = 3.64, *p* = 0.00) (see Table 3). Likewise, the sleep variable “optimum sleep” was analyzed with a Chi-square test, with statistically significant values observed in the control group.

**Figure 2:**
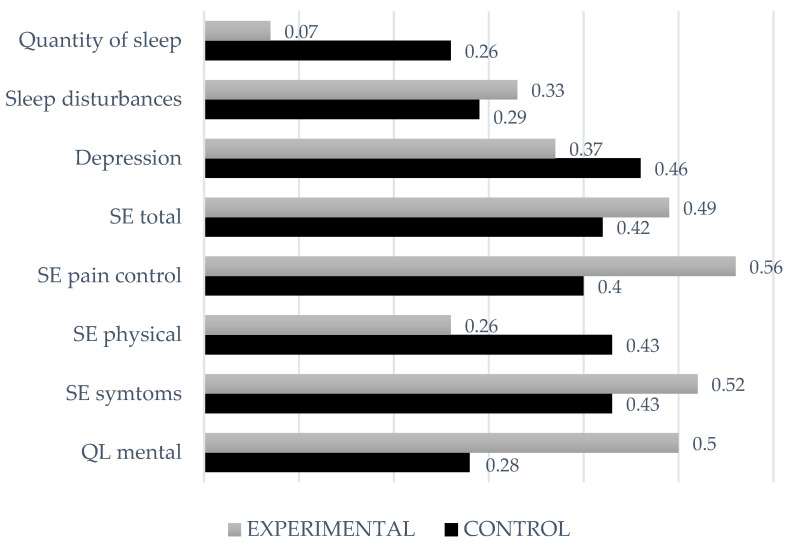
Effect sizes (d) for the experimental and control groups for the variables in which both groups improved significantly.

Lastly, analysis of intragroup differences revealed that 14 of the 18 variables studied improved in the control group, while in the experimental group, improvements were only observed in 10 out of 18 variables. In both groups, improvements were observed in the perception of pain in the past 3 days, subjective quality of health, self-efficacy (in all dimensions), depression, and sleep alterations and quantity. Although the experimental group obtained larger effect sizes in almost all the variables (0.07 < d < 0.56), except for depression and self-efficacy, the control group obtained a larger effect size in the management of physical symptoms (see Figure 2). Ultimately, the effect sizes found in both therapies were small to medium at most (in CBT 0.2 < d < 0.46).

It is necessary to highlight that the control group, aside from showing improvements in these variables, also showed significant improvements at the intragroup level, in the variables: anxiety (*t* = 3.02, *p* =0.00), somnolence (*t* = −3.24, *p* = 0.00), adaptation (*t* = 2.66, *p* = 0.00) and sleep interference (with six and nine items) (SLP 6: *t* = 3.11, *p* = 0.00; SLP 9: *t* = 3.23, *p* = 0.00). For its part, the experimental group, despite having participants with greater levels of pain in the present at the start of treatment, showed statistically lower levels at the end of their sessions (*t* = 2.96, *p* = 0.00).

## 4. Discussion

The present study was conducted to compare two therapies which had obtained good results for chronic pain according to previous literature. The differences found in the pre-treatment phase between both groups were mostly equalized by the post-treatment stage, coinciding with previous literature that described similar efficacies between the two treatments [11,12]. The authors of the present study hypothesized that the mutual improvements observed in both treatments were perhaps due to content that was common to both therapies. In fact, the intragroup analysis revealed similar improvements in pain perceived over the previous three days, subjective quality of life, self-efficacy, depression, and quantity and quality of sleep, in agreement with previous studies on Cognitive Behavioral Therapy [27,28] and Mindfulness [29]. However, the effect size was slightly larger in the experimental group, indicating that even though there were similar improvements in both groups, the greatest change was found in the experimental group. The literature comparing both therapies is scarce, and it is therefore difficult to compare the present study with previous results. Other authors also studied only a few variables in each trial, which rarely coincided with our variables [6]. Thus, the current comparison is considered the main contribution of the present study, as in both therapies a larger number of variables were analyzed compared with previous studies.

Along these lines, the results of this publication provide new evidence on the efficacy of MBCT-CT on the improvement of the variables analyzed, a notable difference to previously mentioned studies which only included a smaller number of variables.

One of the main differences between our groups was the superior effect of MBCT-CP in reducing pain at the present time, as pointed out in previous studies [10,30]. The experimental groups showed a statistically significant level of pain at the start of therapy, with significant changes before and after treatment as shown by the pain scores obtained at the end of therapy, which were similar to the control group; in contrast, the control group did not report significant changes.

Likewise, MBCT-CP seemed to have a greater efficacy in improving subjective quality of life. Although starting scores were similar in both groups, the superiority of MBCT-CP as compared to CBT was observed at the end of therapy. These results are contradictory to previous literature [11], which indicates a similar efficacy. Another previous study pointed out the relationship between pain and subjective quality of health. Therefore, this significant change in pain could be linked to more notable changes in the subjective quality of health. This effect was only observed in the experimental group, as this group reported statistically significant levels of pain improvement [31].

CBT obtained better results in variables associated with sleep, especially adequacy of sleep. Before therapy, the CBT group (control) showed a lower amount of adequate sleep, which significantly improved after treatment, with the result being statistically significant as compared to the experimental group. Likewise, when treatment ended, the quantity and optimum level of sleep were significantly higher in the control group. Previous research [32] indicates a modest superiority of CBT for improving the assessment of sleep as compared to other types of variables, such as those related to pain, with a greater superiority found for Mindfulness. Thus, some authors indicate better results in both types of variables [33] by administering CBT for both insomnia and chronic pain, or by fusing CBT with Mindfulness [34].

These results are partially in agreement with those found in the previous literature, although some limitations were also found. One of the main limitations is the comparability of the groups; since there was no randomization, the diagnoses were not controlled, and the groups were created at different times (possible cohort effects), in addition to the fact that the control group was composed of younger individuals and included more disabled individuals. It is possible that the control sample, with a more disabled population, had a worse response to the treatment due to the interference of pain in daily functioning, or perhaps it was due to the predominant type of diagnosis, whose nature we unfortunately do not know. In addition, it is necessary to point out that previous differences in comorbidities such as anxiety or depression were not explored, which may interfere with the results for these two variables. Lastly, the lack of training of the therapists may have conditioned the results, and the absence of a follow-up could limit the conclusions.

However, the overall validity is still considered strong, as the study took place in clinical contexts and on a miscellaneous sample, thereby allowing for the comparison of therapies, which is difficult to find in the literature.

## 5. Conclusions

We can thus conclude that CBT and Mindfulness-based Cognitive Therapy may have a similar efficacy, although it seems that there was a modest trend of larger effects produced by MBCT-CP. Likewise, CBT seemed to improve variables associated with sleep, while MBCT-CP seemed to be more efficient in the improvement of variables related with pain and quality of life. However, as the comparability of the samples could be questionable, further research along these lines is necessary.

## Figures and Tables

**Table 1:** Content of the experimental and control group sessions.

**Experimental Group: Mindfulness**	**Control Group: Cognitive-Behavioral Therapy**
**1. Abandon the Automatic Habit of Pain**	**Session 1**
Introduction to the program: welcome, presentations. Rules, objectives, roles, and responsibilities. Gate Control Theory to explain the mechanism of pain perception. Introduction to the most common meditation practices.	Introduction to the program: welcome, presentations. Rules, objectives, roles, and responsibilities. Introduction to phase 1 of Jacobson’s progressive muscle relaxation. Explanation of psychological factors of pain.
**2. Facing the Challenges**	**Session 2**
Introduction of the ABC model. Stress–pain thermometer. Pleasant Mindfulness experiences. Breathing meditation.	Abdominal breathing exercises. Self-recording of activities and relationship with pain. Progressive planning of goals.
**3. Breathing as an Anchor**	**Session 3**
Senses meditation. Breathing as an anchor and sitting meditation. Working with unpleasant physical sensations. Awareness of stressful experiences.	Phase 2 of Jacobson’s progressive muscle relaxation. Review of short-term goals and activities. Explanation of compartmentalized health.
**4. Learning to be Present**	**Session 4**
Sitting meditation: mindfulness sounds and thoughts. Diary of stressful experiences and discussion about futile mental habits. Responsive 3 min breathing meditation. Mindfulness movement.	Jacobson review, short-term goals and activities. Submission of self-recording of thoughts. Abdominal breathing and guided imagination.
**5. Active Acceptance**	**Session 5**
Meditation in silence. The process of active acceptance. Automatic thoughts, intermediate beliefs, and main beliefs. Awareness of mental patterns. Sitting meditation.	Phase 3 of Jacobson’s progressive muscle relaxation. Self-recording of thoughts and distraction techniques. Presentation about changes in thoughts and acceptance techniques.
**6. Thoughts as Only Thoughts**	**Session 6**
Tendency towards interpretation. Seeing thoughts only as thoughts. Working on difficulties. Relationship between emotional and physical state and thoughts. Changing point of view. Pain thermometer. Maintenance plan.	Phase 3 of Jacobson’s progressive muscle relaxation. Review of self-recording and distraction techniques. Presentation about acceptance techniques. Guided visual relaxation.
**7. Caring for Oneself**	**Session 7**
Sitting meditation: working on difficult thoughts, training on acceptance without judgement. Identification of signs of alarm and plans to decrease stress. Full attention on daily-life activities. Debate on informal practices.	Rapid phase of Jacobson’s muscular relaxation. Review recording of thoughts and distraction techniques. Brief introduction to self-criticism and re-enforcement exercises.
**8. Maintenance in the Management of Chronic Pain**	**Session 8**
Body scanner. Identification of red flags and coping options. Mindfulness backpack. Maintenance plan. Shell meditation.	Prevention of relapses and re-enforcement of exercise continuity. Evaluation tests.

**Table 2:** Sociodemographic characteristics, medical diagnoses, and participating centers of the experimental and control groups. Differences in frequencies between both groups were determined by Chi-square test.

Variables	Experimental Group	Control Group	Χ^2^	*p*
	*n* (%)	*n* (%)		
Sex				
Male	13 (22.8)	34 (32.4)	1.64	0.2
Female	44 (77.2)	71 (67.6)		
Marital status				
Single	10 (17.6)	13 (12.4)	2.03	0.56
Married	33 (57.9)	75 (71.5)		
Divorced	8 (14)	10 (9.5)		
Widow	6 (10.5)	7 (6.7)		
Occupation				
Employed	11 (19.3)	11 (10.5)	16.98	0.00
On leave	9 (15.8)	28 (26.6)		
Disabled	10 (17.5)	34 (32.4)		
Retired	15 (26.3)	23 (21.9)		
Homemaker	12 (21.1)	9 (8.6)		
Diagnosis				
Lumbago	22 (38.6)	-		
Cervicalgia	7 (12.3)	-		
Fibromyalgia	7 (12.3)	17 (16.2)	0.45	0.5
Rheumatoid arthritis	6 (10.5)	-		
Other medical conditions	15 (26.3)	-		
Center				
General University Hospital of Alicante	23 (40.4)	105 (100)		
Marina Baixa Hospital	11 (19.3)	-		
Vega Baja Hospital	23 (40.4)	-		

**Table 3:** *t*-test results of the differences in pre-treatment and post-treatment means from the control and experimental group. *t*-test differences and effect sizes (d) for intragroup differences in the experimental group and treatment.

	Pre-Treatment			Post-Treatment			Pre–Post Difference	
	Control	Experimental		Control	Experimental		Control	Experimental
	M (SD)	M(SD)	T(*p*)	M (SD)	M(SD)	T(*p*)	T(*p*)*d*	T(*p*)*d*
**Intensity of pain during last 3 days**	6.28 (2.14)	6.54 (1.85)	−1.61 (0.11)	7.02 (2.11)	7.54 (1.56)	−0.76 (0.14)	**3.71 (0.00) 0.42**	**3.61 (0.00) 0.07**
**Intensity of pain at present**	2.56 (0.86)	2.95 (0.65) **	−3.15 (0.00)	2.4 (0.91)	2.6 (0.72)	−1.41 (0.16)	1.61 (0.11)	**2.96 (0.00) 0.47**
**QL physical**	28.60 (5.62)	28.54 (2.31)	0.46 (0.68)	30.22 (8.11)	28.54 (8.31)	1.17 (0.24)	−0.94 (0.34)	0.05 (0.96)
**QL mental**	31.84 (10.96)	35.15 (11.91)	−0.78 (0.44)	31.97 (11.34)	38.96 (13.26) **	−3.34 (0.00)	**2.03 (0.04) 0.28**	**2.98 (0.00) 0.5**
**SE symptoms**	35.07 (14.96)	32 (12.71)	1.31 (0.19)	41.51 (16.70)	40.33 (17.96)	0.41 (0.67)	**−4.88 (0.00) 0.43**	**−4.01 (0.00) 0.52**
**SE physical**	27.26 (14.64)	25.21 (13.91)	0.86 (0.38)	32.6 (14.88)	29 (14.7)	1.47 (0.14)	**−4.85 (0.00) 0.43**	**−1.93 (0.06) 0.26**
**SE control pain**	**15.45 (10.96)** *	11.98 (9.64)	2 (0.04)	17.54 (11.92)	18.14 (12.04)	−1.43 (0.15)	**−4.51 (0.00) 0.40**	**−4.26 (0.00) 0.56**
**SE total**	77.80 (36.02)	69.19 (31.54)	1.51 (0.13)	89.57 (35.84)	86.85 (41.22)	0.43 (0.66)	**−5.64 (0.00) 0.42**	**−3.78 (0.00) 0.49**
**Anxiety**	11.61 (4.78)	10.94 (4.06)	0.88 (0.37)	10.37 (4.95)	10.8 (4.11)	0.36 (0.71)	**3.02 (0.00) 0.28**	1.78 (0.08) *-*
**Depression**	10.73 (4.80)	11.15 (9.36)	−0.54 (0.59)	8.93 (4.94)	9.36 (4.79)	−0.54 (0.59)	**5.26 (0.00) 0.46**	**3.12 (0.00) 0.37**
**Sleep alterations**	61.97 (26.36) **	30.95 (15.61)	8.12 (0.00)	55.69 (27.27)	58.83 (25)	−0.72 (0.47)	**3.07 (0.00) 0.29**	**−8.12 (0.00) 0.33**
**Quantity of sleep**	5.38 (1.44)	5.28 (1.36)	0.59 (0.55)	5.94 (1.56) **	5.29 (1.35)	2.53 (0.01)	**−2.74 (0.00) 0.26**	**−8.12 (0.00) 0.07**
**Snoring**	46.53 (32.67)	56.42 (38.91)	−1.61 (0.11)	43.42 (31.43)	51.63 (36.2)	−1.47 (0.14)	1.04 (0.29)	1.19 (0.24) -
**Waking up**	46.07 (34.09)	45.35 (31.33)	−1.21 (0.23)	39.23 (29.86)	44.56 (31.62)	−1.06 (0.29)	0.27 (0.78)	0.21(0.84) -
**Somnolence**	46.90 (24.78) **	45.38 (23.10)	−3.91 (0.00)	41.80 (31.24)	45.38 (23)	−0.75 (0.45)	**−3.24 (0.00) 0.30**	0.54 (0.59) -
**Adequacy**	50.06 (24.14) **	30 (25.14)	4.51 (0.00)	44.69 (24.21) **	30 (25.01)	3.64 (0.00)	**2.66 (0.00) 0.25**	0.19 (0.85) -
**Sleep interference** **6 items**	59.76 (21.64)	57.42 (21.14)	−0.87 (0.39)	50.76 (23.91)	51.42 (21.14)	−1.76 (0.08)	**3.11 (0.00) 0.29**	0.83 (0.41) -
**Sleep interference** **9 items**	60.25 (21.28)	56.16 (21.05)	−0.79 (0.43)	51.90 (23.07)	56.16 (21.05)	−1.15 (0.25)	**3.23 (0.00) 0.30**	1.42 (0.16) -

Values in **bold** indicate statistically significant differences. * *p* ≤ 0.05, ** *p* ≤ 0.01; QL = Quality of life; SE = Self-efficacy.

## Data Availability

The data presented in this study are available on request from the corresponding author. The data are not publicly available due them being unpublished results.
